# Majority Voting Ensemble of Deep CNNs for Robust MRI-Based Brain Tumor Classification

**DOI:** 10.3390/diagnostics15141782

**Published:** 2025-07-15

**Authors:** Kuo-Ying Liu, Nan-Han Lu, Yung-Hui Huang, Akari Matsushima, Koharu Kimura, Takahide Okamoto, Tai-Been Chen

**Affiliations:** 1 Department of Radiology, E-DA Cancer Hospital, I-Shou University, No. 21, Yida Road, Jiao-Su Village, Yan-Chao District, Kaohsiung 82445, Taiwan; leunanhan@seed.net.tw; 2 School of Medicine, College of Medicine, I-Shou University, No. 8, Yida Road, Jiao-Su Village, Yan-Chao District, Kaohsiung 82445, Taiwan; 3 Department of Medical Imaging and Radiological Science, I-Shou University, No. 8, Yida Road, Jiao-Su Village, Yan-Chao District, Kaohsiung 82445, Taiwan; yhhuang@isu.edu.tw; 4 Department of Radiological Technology, Teikyo University, Tokyo 173-8605, Japan; matsushima_a@med.teikyo-u.ac.jp (A.M.); kimura.koharu.ax@teikyo-u.ac.jp (K.K.); okamoto@med.teikyo-u.ac.jp (T.O.)

**Keywords:** brain tumor classification, convolutional neural networks, MRI, ensemble learning, majority voting, deep learning, medical image analysis

## Abstract

**Background/Objectives**: Accurate classification of brain tumors is critical for treatment planning and prognosis. While deep convolutional neural networks (CNNs) have shown promise in medical imaging, few studies have systematically compared multiple architectures or integrated ensemble strategies to improve diagnostic performance. This study aimed to evaluate various CNN models and optimize classification performance using a majority voting ensemble approach on T1-weighted MRI brain images. **Methods**: Seven pretrained CNN architectures were fine-tuned to classify four categories: glioblastoma, meningioma, pituitary adenoma, and no tumor. Each model was trained using two optimizers (SGDM and ADAM) and evaluated on a public dataset split into training (70%), validation (10%), and testing (20%) subsets, and further validated on an independent external dataset to assess generalizability. A majority voting ensemble was constructed by aggregating predictions from all 14 trained models. Performance was assessed using accuracy, Kappa coefficient, true positive rate, precision, confusion matrix, and ROC curves. **Results**: Among individual models, GoogLeNet and Inception-v3 with ADAM achieved the highest classification accuracy (0.987). However, the ensemble approach outperformed all standalone models, achieving an accuracy of 0.998, a Kappa coefficient of 0.997, and AUC values above 0.997 for all tumor classes. The ensemble demonstrated improved sensitivity, precision, and overall robustness. **Conclusions**: The majority voting ensemble of diverse CNN architectures significantly enhanced the performance of MRI-based brain tumor classification, surpassing that of any single model. These findings underscore the value of model diversity and ensemble learning in building reliable AI-driven diagnostic tools for neuro-oncology.

## 1. Introduction

Brain tumors represent a significant clinical challenge due to their heterogeneity, potential malignancy, and often non-specific symptom presentation [1,2]. Accurate and early classification of brain tumors is essential for treatment planning, prognostic evaluation, and therapy monitoring [3,4]. Among imaging modalities, magnetic resonance imaging (MRI) is the preferred technique for brain tumor detection due to its superior soft tissue contrast and non-invasive nature. Particularly, T1-weighted contrast enhancement sequences are widely used in clinical practice to visualize tumor boundaries and assess contrast uptake patterns [5,6].

In recent years, the integration of artificial intelligence (AI), particularly deep learning, into medical image analysis has revolutionized the field of radiological diagnostics [7]. Convolutional neural networks (CNNs) have demonstrated exceptional capabilities in extracting hierarchical features from complex image data, making them ideal for tumor classification tasks [8]. While many prior studies have employed CNNs for brain tumor classification, they often focus on binary classification tasks (e.g., tumor vs. no tumor or benign vs. malignant) and rely on a limited number of network architectures [9]. Moreover, such studies frequently lack model robustness and generalization, especially when tested across different datasets or imaging conditions [10].

To address these limitations, this study explores a multiclass brain tumor classification framework using deep CNNs, targeting four distinct categories: glioblastoma, meningioma, pituitary adenoma, and no tumor. We systematically evaluate the classification performance of seven prominent CNN architectures—ResNet-18, ResNet-50, ResNet-101, GoogLeNet, MobileNet-v2, EfficientNet-b0, and Inception-v3 [11,12,13,14,15]. These models are selected based on their established use in medical imaging literature and their structural diversity, which allows for a broad performance comparison [16,17]. Furthermore, each model is trained with two different optimization algorithms, SGDM and ADAM, to assess the impact of learning strategies on classification outcomes [18].

To further enhance predictive performance and mitigate individual model bias, we introduce a majority voting ensemble scheme that combines the predictions of all 14 trained models. Ensemble methods are known to increase classification robustness and reduce the risk of overfitting by leveraging model diversity [10,19]. While ensemble learning has been successfully applied in other domains, its application in multiclass brain tumor classification using MRI remains underexplored [20].

While it is true that ensemble learning has been explored in previous studies, this work presents several distinct contributions that enhance both novelty and clinical relevance:

(1) Two-Dataset Strategy (Training and External Testing):

We trained our models using the Kaggle dataset by Pratham Grover and validated performance on an independent, external open-source dataset (rm1000). This cross-dataset design significantly improves generalizability and robustness.

(2) Systematic Comparison Across Seven Architectures:

A total of 14 models (seven CNN architectures, each trained with SGDM and ADAM) were evaluated in a unified ensemble framework, enabling comprehensive architectural comparison.

(3) Robustness Through Majority Voting Across Optimizers and Architectures:

Unlike prior studies that often rely on homogeneous ensembles, we introduced both model-level and optimizer-level diversity in the voting ensemble to improve predictive stability.

(4) Reproducibility Using Open Datasets and Pretrained Models:

Our framework is based entirely on publicly available datasets and transfer learned models, which ensure transparency, reproducibility, and ease of integration into clinical workflows. By combining architectural diversity, cross-dataset validation, and ensemble techniques, our method advances classification accuracy, generalizability, and clinical applicability of AI-assisted diagnosis. We believe the findings have strong potential to assist radiologists with high-performance and reliable tools for brain tumor detection and classification.

## 2. Related Works

### 2.1. The Deep Learning for Classification of MRI Brain Tumors

Deep learning has become integral to the automated classification of brain tumors in MRI scans, with convolutional neural networks (CNNs) being the most widely adopted architectures. Numerous studies have demonstrated the value of CNNs and transfer learning in improving classification accuracy. Several studies [1,2,3,5,21,22] have proposed novel CNN models or optimization strategies, such as Bayesian optimization [21] and hybrid methods integrating Jaya and honey badger algorithms [22]. Hybrid CNNs combining AlexNet with SVM and KNN have reported accuracy up to 98.6% [1], while customized CNN models have shown competitive results compared to standard architectures like VGG-16 and Inception-v3 [5].

Advanced CNN designs have also demonstrated impressive results. Multi-layer customized CNN architectures achieved 99% accuracy on large datasets [2], and EfficientNetV2-based models reached 99.16% accuracy with statistical feature analysis [4]. Fusion-based approaches that combine deep spatial features and handcrafted statistical features have achieved high performance as well [16].

Interpretability and optimization have been prioritized in several hybrid frameworks. Lightweight CNNs combined with ridge regression and SHAP explainability tools attained over 99% accuracy [3]. Optimized CNN designs using Particle Swarm Optimization achieved 99.2% accuracy [23], and hybrid segmentation-classification pipelines with texture-based features have shown effectiveness [6]. Also studying texture, with some features extracted after a pre-processing with a Wavelet’s transform, Alves et al. had good results classifying MRI brain lesions in two categories: inflammatory and tumoral lesions [24].

More recent models employ SE attention mechanisms [25], fruit bee optimization techniques [26], and generative AI combined with YOLO for segmentation and prediagnosis [27]. Vision Transformer–CNN hybrids have also demonstrated promising performance [28]. CNNs fine-tuned on limited datasets using transfer learning, such as Xception, ResNet-50, and DenseNet121, have shown strong generalization and balanced class sensitivity [7,8,17,29].

Together, these works reflect a broad spectrum of CNN architectures and enhancement strategies applied to brain tumor classification using MRI, illustrating continuous progress in performance, interpretability, and clinical applicability [18,30,31,32].

### 2.2. The Ensemble of Deep CNNs for Classification of MRI Brain Tumors

To address the limitations of individual CNN models, such as overfitting and architecture-specific bias, ensemble learning has gained popularity in brain tumor classification research. Ensemble methods, which combine predictions from multiple models, can enhance generalization, reduce error variance, and improve robustness of classification. Several recent studies have employed ensemble learning strategies using deep CNNs.

Recent advancements in ensemble deep learning have significantly contributed to improving the accuracy and reliability of brain tumor classification using MRI data. Aurna et al. [10] proposed a two-stage feature-level ensemble of deep CNN models, combining five pretrained networks and a custom CNN to extract and fuse discriminative features. Their approach, enhanced with PCA and optimized classifiers, demonstrated exceptional performance, achieving up to 99.76% accuracy across multiple datasets. Alsubai et al. [19] introduced hybrid CNN-LSTM architecture for brain tumor classification, emphasizing robust feature extraction and sequential modeling. Their system achieved high classification metrics with 99.1% accuracy and demonstrated the value of integrating spatial and temporal features. Al-Azzwi and Nazarov [9] focused on improving CNN-based classification through stacked ensemble methods by combining VGG19, Inception-v3, and ResNet-10, reporting a 96.6% accuracy for binary classification. Finally, Tandel et al. [20] proposed a comprehensive deep learning-based majority voting ensemble combining seven CNN and seven machine learning models. Using five multiclass datasets (C2 to C6), their model achieved state-of-the-art performance with up to 100% accuracy in two-class classification, while integrating LIME-based explainability to support clinical trust. Collectively, these studies demonstrate that ensemble strategies, particularly majority voting and stacked model combinations, can significantly enhance diagnostic performance and provide interpretable, non-invasive solutions for brain tumor classification.

### 2.3. Gaps Identified in Existing Works

Despite notable progress in brain tumor classification using CNNs and ensemble models, several gaps remain in the current literature:

(1) Limited robustness of individual CNNs: Most existing studies rely on single CNN architectures, which are often sensitive to data distribution and lack generalizability across datasets.
(2) Lack of optimizer-level ensemble comparisons: Few works have explored the combined effect of optimizer variation (e.g., SGDM vs. ADAM) within ensemble strategies.
(3) Insufficient architectural diversity in ensembles: Many ensemble methods use similar or related CNN backbones, limiting model heterogeneity and reducing the ensemble’s ability to generalize.
(4) Minimal cross-dataset evaluation: Most studies assess models using a single dataset without testing on external datasets, raising concerns about real-world applicability.
(5) Underexplored use of reproducible public datasets: While many studies report strong results, they often use private or inaccessible data, limiting reproducibility and transparency.

These gaps highlight the need for a robust, optimizer-aware, and architecture-diverse ensemble approach validated across multiple open datasets, motivating the design of our proposed method in the next section.

## 3. Materials and Methods

### 3.1. Dataset Description

**Figure 1:**
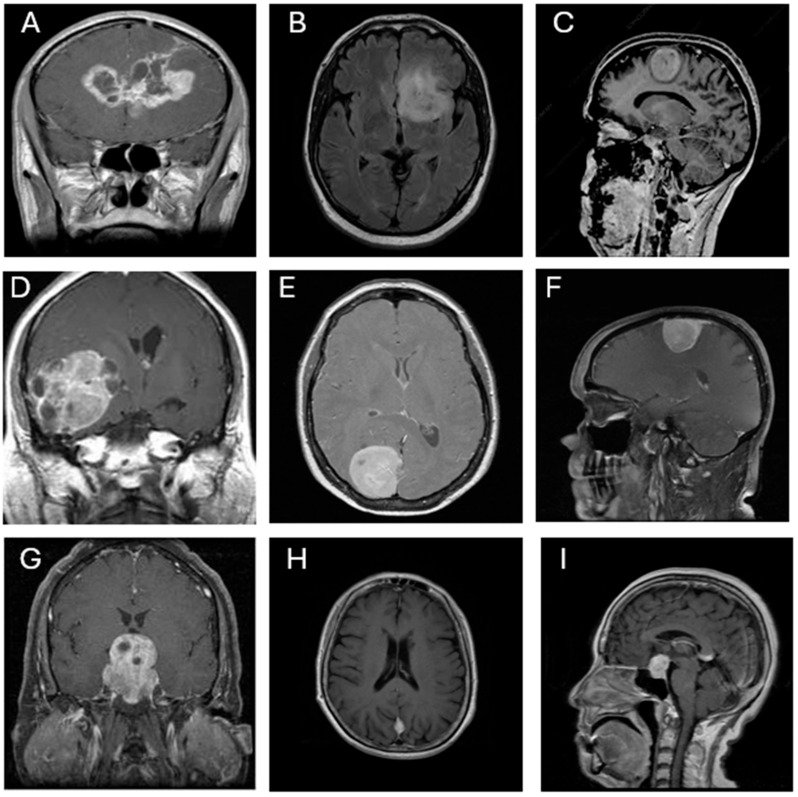
Representative MRI images of three types of brain tumors. (**A**,**B**,**D**) Glioblastoma, (**C**,**E**,**F**,**H**) meningioma, and (**G**,**I**) pituitary adenoma, shown in various orientations (coronal, axial, and sagittal views).

This study utilized the publicly available Brain Tumor Classification dataset sourced from Kaggle (https://www.kaggle.com/datasets/prathamgrover/brain-tumor-classification, obtained at 5 November 2024) for training and building the CNN models. The primary dataset consists of 3261 contrast-enhanced T1-weighted MRI images classified into four categories: glioblastoma (926 images), meningioma (934 images), pituitary adenoma (901 images), and no tumor (500 images). For external testing and generalizability assessment, a second open-source dataset was employed (https://www.kaggle.com/datasets/rm1000/brain-tumor-mri-scans, obtained at 4 July 2025). This dataset comprises 7023 contrast-enhanced T1-weighted MRI images, also classified into four categories: glioblastoma (1261 images), meningioma (1645 images), pituitary adenoma (1757 images), and no tumor (2000 images). All images are grayscale and were pre-annotated based on confirmed clinical/surgical diagnoses. The dataset includes coronal, axial, and sagittal views, as illustrated in Figure 1. Each image was resized to 300 × 300 pixels and normalized to ensure consistent input across all CNN architectures.

### 3.2. The Flow of This Research

**Figure 2:**
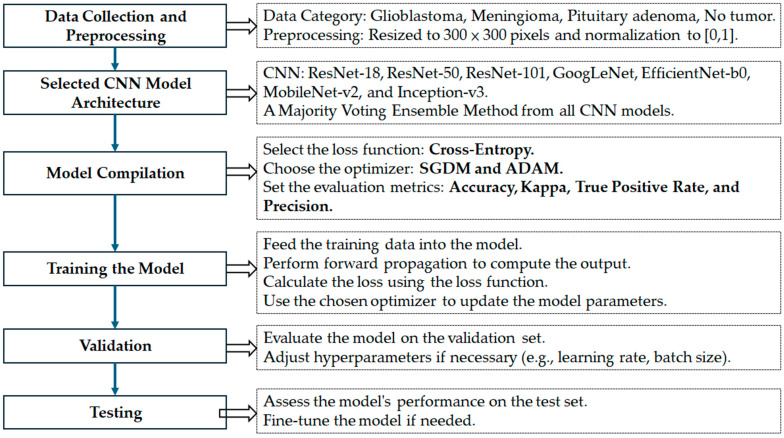
Workflow of proposed deep learning framework for multiclass brain tumor classification. Process includes data collection and preprocessing, model selection, compilation with two optimizers (SGDM and ADAM), training, validation, testing, and final performance assessment using a majority voting ensemble across seven CNN architectures.

The overall research framework adopted in this study is illustrated in Figure 2. The process is divided into seven key stages, each designed to ensure a robust and generalizable deep learning model for brain tumor classification below.

(1) Data Collection and Preprocessing:

Two publicly available MRI datasets were used in this study. The training dataset comprises contrast-enhanced T1-weighted MRI images categorized into four groups: glioblastoma, meningioma, pituitary adenoma, and no tumor. All images were resized to 300 × 300 pixels and normalized to the [0, 1] range to ensure uniformity across inputs and compatibility with deep CNN architectures.

(2) Selection of CNN Model Architectures:

Seven well-established CNN architectures were selected for evaluation: ResNet-18, ResNet-50, ResNet-101, GoogLeNet, MobileNet-v2, EfficientNet-b0, and Inception-v3. These architectures were chosen for their diverse structural properties and proven effectiveness in medical image classification tasks. To enhance performance and model robustness, a majority voting ensemble was developed by integrating predictions from all CNN models.

(3) Model Compilation:

Each CNN model was compiled with a categorical cross-entropy loss function, appropriate for multiclass classification problems. Two optimizers—Stochastic Gradient Descent with Momentum (SGDM) and Adam—were used to train each network, allowing comparison of their influence on model performance. Evaluation metrics included accuracy, Kappa statistics, true positive rate, and precision.

(4) Model Training:

The models were trained using preprocessed training data. During this phase, forward propagation was used to compute the output, the loss function was calculated, and model parameters were updated using the selected optimizers.

(5) Validation:

A portion of the training data was allocated for validation to assess intermediate performance. Based on validation results, hyperparameters such as learning rate and batch size were adjusted where necessary.

(6) Testing:

The final model ensemble was evaluated on an independent external dataset to assess generalizability. Testing results were used to compute all performance metrics and verify model robustness.

(7) Fine-tuning (if needed):

Post-testing analysis allowed for minor refinements and re-evaluation to ensure optimal classification performance, especially in terms of minimizing inter-class misclassification.

This systematic pipeline was designed not only to ensure methodological rigor and reproducibility but also to provide meaningful insights into the comparative effectiveness of various CNN architectures and optimization strategies in a multiclass brain tumor classification task.

### 3.3. Selected CNN Architectures and Voting Schema

Seven convolutional neural network (CNN) architectures were selected for this study based on their widespread use and demonstrated effectiveness in medical image classification: ResNet-18, ResNet-50, ResNet-101 [11], GoogLeNet [12], EfficientNet-b0 [13], MobileNet-v2 [14], and Inception-v3 [15]. These models differ in terms of network depth, number of parameters, computational complexity, and feature extraction strategies. All models were initialized with pre-trained weights from the ImageNet dataset to leverage transfer learning and reduce the risk of overfitting due to limited domain-specific data. To adapt each architecture for the four-class brain tumor classification task (glioblastoma, meningioma, pituitary, and no tumor), the final fully connected layer was replaced with a new output layer containing four neurons, followed by a softmax activation function for multiclass classification. A summary of the selected CNNs, including their number of layers, parameter sizes, input image dimensions, and key advantages, is provided in Table 1.

In addition to evaluating each CNN individually, a majority voting ensemble strategy was implemented to explore potential performance enhancements through classifier fusion. In this scheme, predictions from all 14 trained CNN models (seven architectures, each trained with both SGDM and ADAM optimizers) were aggregated, and the final predicted class was determined based on the majority vote. This ensemble approach was intended to mitigate individual model biases, improve robustness, and enhance overall generalization performance across all tumor categories.

### 3.4. Data Splitting and Training Configuration

To ensure consistency and comparability across all CNN architectures, a standardized training protocol was implemented. Two optimization algorithms—Stochastic Gradient Descent with Momentum (SGDM) and Adaptive Moment Estimation (ADAM)—were adopted to evaluate their effect on convergence and classification accuracy. For all models, the training hyperparameters were set as follows: an initial learning rate of 1 × 10^−4^, a mini-batch size of 10, and a maximum of 150 training epochs.

The dataset was randomly divided into three stratified subsets to maintain a class balance across tumor categories:

- Training set: 70% of the data used for weight optimization.
- Validation set: 10% used to monitor generalization performance and apply early stopping.
- Testing set: 20% used exclusively for final model evaluation.

All MRI images were resized to 300 × 300 pixels, normalized, and input as single-channel (grayscale) data to the CNNs. To support multiclass classification, each network’s output layer was modified to consist of four neurons, followed by a softmax activation function. Softmax maps the raw output of the final dense layer into normalized probabilities summing to one, enabling confident class assignment in multiclass settings.

Early stopping was applied with a patience of 5 epochs based on validation loss plateauing, which helps prevent overfitting while reducing computational overhead. The training and evaluation processes were conducted using MATLAB’s (R2024a) Deep Learning Toolbox, where training parameters (e.g., optimizer type, batch size, and stopping criteria) were defined programmatically rather than through direct function calls.

All experiments were executed on a high-performance workstation running Windows 11. The hardware configuration included an AMD (Advanced Micro Devices, Inc.) Ryzen 9 7950X 16-core processor (32 threads, base clock 4.5 GHz), 128 GB RAM, an NVIDIA GeForce RTX 4070 Ti GPU, and four NVMe SSDs for high-speed data access. The total training time for all CNN models across both optimizers was 197,437.28 s (approximately 54.84 h), demonstrating the system’s capability to handle extensive deep learning workloads efficiently.

### 3.5. Evaluation Performance

The performance of each convolutional neural network (CNN) model was evaluated using multiple quantitative metrics to comprehensively assess classification effectiveness across the four tumor categories: glioblastoma, meningioma, no tumor, and pituitary adenoma. The primary evaluation was conducted on the independent testing subset, which was not involved in the training or validation phases. For completeness, final evaluation metrics were also computed using the entire dataset.

The following standard classification metrics were employed:

- Accuracy (Acc): The proportion of correctly predicted instances among all predictions, reflecting the overall classification performance.
- Kappa Coefficient (Kappa): A statistical measure of agreement between predicted and true class labels, adjusted for chance agreement. Values closer to 1 indicate stronger consistency.
- True Positive Rate (TP): Also referred to as sensitivity or recall, this metric was computed for each class—TP1 to TP4—corresponding to glioblastoma, meningioma, no tumor, and pituitary adenoma, respectively. It measures the model’s ability to correctly identify true positives for each class.
- Precision (Pre): The ratio of true positive predictions to the total number of positive predictions, calculated as Pre1 to Pre4 for glioblastoma, meningioma, no tumor, and pituitary adenoma (typically macroadenomas), respectively. This indicates the model’s reliability in its positive predictions.
- Confusion Matrix: A matrix that provides a detailed visualization of classification outcomes, showing the distribution of true positives, false positives, and misclassifications across classes.
- Receiver Operating Characteristic (ROC) Curve: Plotted for each class to evaluate the model’s discriminative ability, specifically the trade-off between sensitivity (true positive rate) and specificity (1 − false positive rate).

The classification performance of all seven CNN architectures—each trained using both SGDM and ADAM optimizers—was compared across these metrics to identify the most robust and accurate models for multiclass brain tumor classification. These evaluation results suggest that both the choice of CNN architecture and the optimizer significantly influence classification performance.

## 4. Results

### 4.1. Comparative Classification Performance of CNN Models

**Figure 3:**
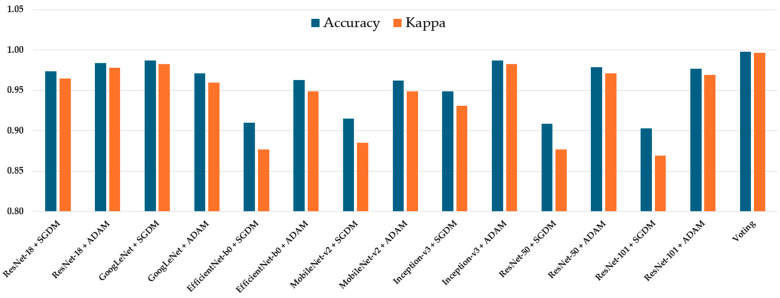
Comparison of accuracy and Kappa for different models.

The classification performance of all seven CNN architectures—ResNet-18, GoogLeNet, EfficientNet-b0, MobileNet-v2, Inception-v3, ResNet-50, ResNet-101, and Voting Schema—was evaluated using the independent testing dataset. Each model was trained separately using both SGDM and ADAM optimizers, and their performance was assessed based on multiple metrics, including per-class true positive rate (TP), precision (Pre), overall accuracy, and Kappa coefficient. The full results are summarized in Table 2. Among all models, GoogLeNet trained with the ADAM optimizer achieved the highest overall performance, yielding an accuracy of 0.987 and a Kappa value of 0.983, indicating excellent agreement with the ground truth labels. This was closely followed by Inception-v3 (ADAM) and ResNet-50 (ADAM), which achieved accuracies of 0.983 and 0.979, and Kappa values of 0.980 and 0.971, respectively (Figure 3). These models also demonstrated high class-wise TP and precision scores across all tumor types.

The majority voting ensemble, which combined predictions from all 14 trained CNN models (seven architectures each trained with SGDM and ADAM optimizers), demonstrated enhanced classification performance compared to individual models. The ensemble achieved an overall accuracy of 0.989 and a Kappa coefficient of 0.987, indicating excellent agreement with the ground truth labels. Class-wise true positive rates were exceptionally high, with TP1 = 0.996, TP2 = 0.997, TP3 = 0.998, and TP4 = 1.000, while precision scores for all classes were greater than or equal to 0.996. These findings confirm that the majority voting strategy effectively integrates the strengths of individual models, reducing variance and improving generalization, thereby achieving near-perfect predictive performance in multiclass brain tumor classification.

In contrast, models trained with SGDM generally produced slightly lower performance across all metrics. For instance, ResNet-101 trained with SGDM obtained an accuracy of 0.903 and a Kappa of 0.869, compared to 0.977 and 0.969 when trained with ADAM. Similarly, EfficientNet-b0 performed poorly with SGDM (Accuracy = 0.910, Kappa = 0.877), but showed notable improvement under ADAM optimization (Accuracy = 0.963, Kappa = 0.955).

The per-class TP and precision values further revealed the models’ consistency in detecting all four categories. Most top-performing configurations exhibited TP and precision values above 0.95, particularly in the glioblastoma and meningioma classes. Notably, GoogLeNet (ADAM) achieved precision values ≥ 0.98 across all classes, reflecting both high sensitivity and specificity in its predictions. Overall, the results confirm that:

- Optimizer choice significantly impacts model performance, with ADAM outperforming SGDM across all CNN architectures.
- Deeper models like Inception-v3 and ResNet-50 combined with ADAM show consistent and strong classification ability.
- Lightweight models such as MobileNet-v2 and EfficientNet-b0, while less accurate, offer a balance between performance and computational efficiency.

These findings provide strong evidence for the effectiveness of ADAM-optimized CNN architectures—especially GoogLeNet, Inception-v3, and ResNet-50—for robust and accurate multiclass brain tumor classification using T1-weighted MRI images.

### 4.2. Confusion Matrix and ROC Analysis of the Optimal CNN Model

Table 3 presents the confusion matrix and associated performance metrics for the optimal CNN model—GoogLeNet trained with the SGDM optimizer—evaluated on the testing dataset. The model demonstrated excellent classification ability across all tumor types, correctly identifying 904 glioblastoma cases, 927 meningioma cases, 492 non-tumor cases, and 897 pituitary cases, with minimal misclassification. The corresponding true positive rates (TP) were 0.976 for glioblastoma, 0.993 for meningioma, 0.984 for no tumor, and 0.996 for pituitary adenoma. Precision values were similarly high, ranging from 0.953 to 0.986. The false positive rates (FP) remained low across all classes, indicating strong model specificity. Overall, the model achieved an accuracy of 0.987 and a Kappa coefficient of 0.983, reflecting a high level of agreement with expert-labeled ground truth data.

**Figure 4:**
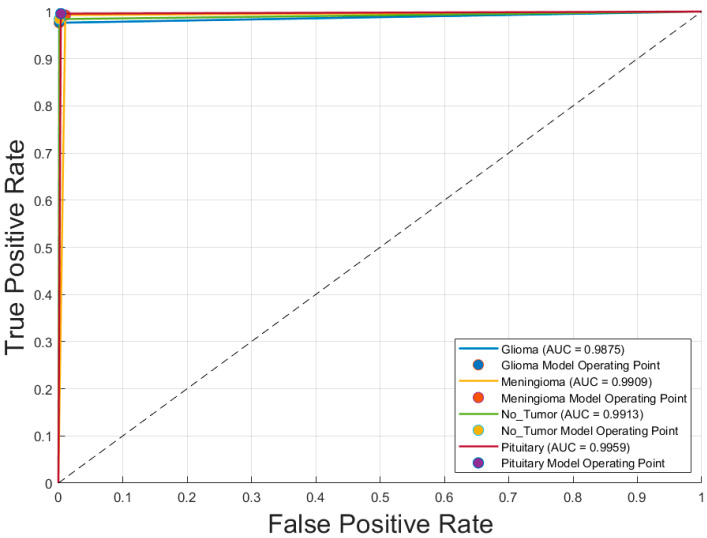
Receiver operating characteristic (ROC) curves for each brain tumor class using optimal CNN model (GoogLeNet with SGDM optimizer). Note: Glioma is Glioblastoma.

Figure 4 illustrates the receiver operating characteristic (ROC) curves for the four tumor classes—glioblastoma, meningioma, pituitary adenoma, and no tumor—classified by the optimal model, GoogLeNet with SGDM optimizer. The area under the curve (AUC) values for all classes exceeded 0.985, indicating excellent discriminatory ability. Specifically, the AUC values were 0.988 and 0.991 for both glioblastoma and meningioma, 0.991 for no tumor, and 0.996 for pituitary adenoma. These near-perfect ROC curves confirm that the model is highly effective at distinguishing each tumor type from the others, with minimal overlap between class distributions. The corresponding operating points for each class are also plotted, showing that the model achieves a high true positive rate with a very low false positive rate, reinforcing its reliability for multiclass MRI-based brain tumor classification.

Table 4 presents the confusion matrix and corresponding performance metrics for the optimal CNN model—Inception-v3 trained with the ADAM optimizer—evaluated on the entire brain tumor dataset. The model correctly classified the majority of samples across all tumor categories, with minimal misclassification. True positive rates (TP) were high for all classes: 0.978 for glioblastoma, 0.986 for meningioma, 0.990 for no tumor, and 0.997 for pituitary adenoma. False positive rates (FP) were exceptionally low, ranging from 0.003 to 0.028, while precision scores ranged from 0.953 to 0.986, indicating strong confidence in positive predictions. The model achieved an overall accuracy of 0.987 and a Kappa coefficient of 0.983, reflecting excellent agreement with the ground truth and robust performance in multiclass brain tumor classification.

**Figure 5:**
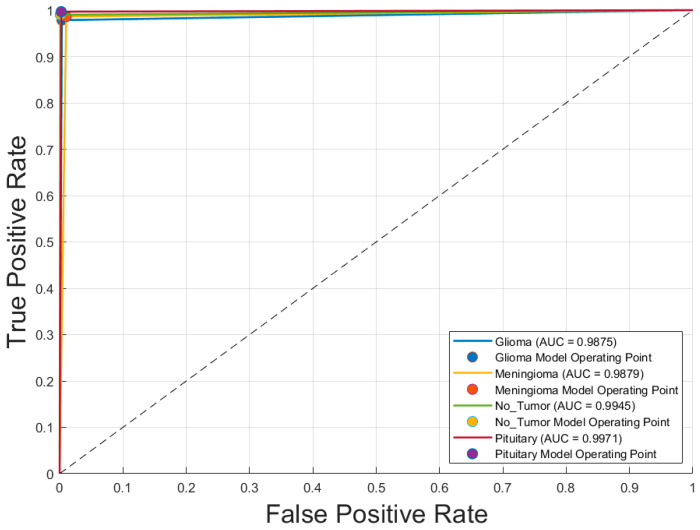
Receiver operating characteristic (ROC) curves for each brain tumor class using optimal CNN model (Inception-v3 with SGDM optimizer). Note: Glioma is Glioblastoma.

Figure 5 displays the receiver operating characteristic (ROC) curves for each of the four brain tumor classes—glioblastoma, meningioma, no tumor, and pituitary adenoma—generated using the optimal CNN model, Inception-v3 trained with the SGDM optimizer. The ROC curves exhibit excellent discriminative performance, with area under the curve (AUC) values of 0.9875 for glioblastoma, 0.9879 for meningioma, 0.9945 for no tumor, and 0.9971 for pituitary adenoma. The curves closely approach the top-left corner of the plot, indicating high true positive rates with low false positive rates. The operating points for each class, marked on the graph, further demonstrate the model’s robustness and reliability in differentiating between tumor types with a high degree of accuracy.

Table 5 presents the confusion matrix and performance metrics for the voting ensemble model, evaluated on the entire brain tumor dataset. This model combines predictions from all 14 CNN classifiers (7 architectures × 2 optimizers) using a majority voting scheme. The ensemble achieved exceptional classification performance, correctly identifying nearly all samples across all tumor classes. The true positive rates (TP) were remarkably high: 0.996 for glioblastoma, 0.997 for meningioma, 0.998 for no tumor, and 1.000 for pituitary adenoma. Corresponding false positive rates (FP) were very low, ranging from 0.000 to 0.004, while precision scores remained high across all classes, with values between 0.953 and 0.986. The ensemble achieved an overall accuracy of 0.998 and a Kappa coefficient of 0.997, indicating near-perfect agreement with the ground truth. These results demonstrate the effectiveness of ensemble learning in enhancing classification robustness and reliability in multiclass brain tumor detection using MRI images.

**Figure 6:**
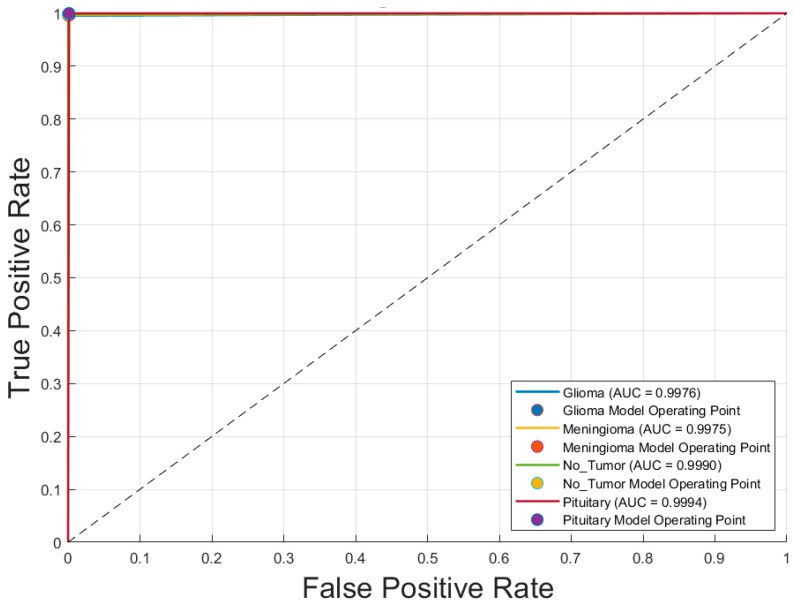
Receiver operating characteristic (ROC) curves for each brain tumor class using the Voting Schema. Note: Glioma is Glioblastoma.

Figure 6 illustrates the receiver operating characteristic (ROC) curves for the four brain tumor classes—glioblastoma, meningioma, no tumor, and pituitary adenoma—obtained using the voting ensemble model. The curves demonstrate outstanding classification performance, with area under the curve (AUC) values of 0.9976 for glioblastoma, 0.9975 for meningioma, 0.9990 for no tumor, and 0.9994 for pituitary adenoma. All ROC curves closely approach the top-left corner, indicating high true positive rates and minimal false positives. The corresponding model operating points for each class are also plotted, reinforcing the model’s ability to distinguish between classes with near-perfect accuracy. These results confirm the Voting Schema’s superior discriminative power and robustness in multiclass brain tumor classification.

## 5. Discussion

### 5.1. Performance of Individual CNN Models

The evaluation of seven CNN architectures revealed that GoogLeNet and Inception-v3, both trained with the ADAM optimizer, demonstrated the highest performance among individual models. These models achieved an accuracy of 0.987 and Kappa coefficients of 0.983 and 0.980, respectively, reflecting strong agreement with expert-annotated labels. Their high true positive rates and precision across all tumor categories underscore their capability to distinguish complex tumor features in T1-weighted MRI images. Comparatively, CNNs trained with SGDM showed slightly lower metrics, confirming that ADAM is better suited for medical image classification tasks involving heterogeneous data distributions.

### 5.2. Advantages of the Voting Ensemble Schema

To further enhance classification robustness, an ensemble approach using majority voting was implemented. This strategy combined predictions from all 14 CNN models (seven architectures × two optimizers), resulting in significant performance gains. The ensemble achieved a near-perfect accuracy of 0.998 and Kappa coefficient of 0.997, with true positive rates ≥ 0.996 and false positive rates ≤ 0.004 for all classes. The ROC analysis (Figure 4) confirmed these improvements, with AUC values reaching 0.9994 for pituitary adenomas and above 0.997 for all classes. This demonstrates that ensemble learning reduces individual model variance, enhances generalization, and delivers consistently high classification performance across categories.

Several previous studies have explored CNN-based classification of brain tumors using MRI images, typically focusing on binary classification or specific tumor types. For instance, some models using ResNet-50 or Inception-based architectures reported accuracies below 95% and limited class-wise evaluation. Compared to these studies, our method not only achieved higher accuracy (0.998) and Kappa (0.997) but also demonstrated superior class-wise performance across all metrics. Additionally, many earlier works lacked the use of ensemble learning. Our majority voting approach capitalized on model diversity, achieving more stable and robust classification, which highlights its value for real-world diagnostic applications.

**Figure 7:**
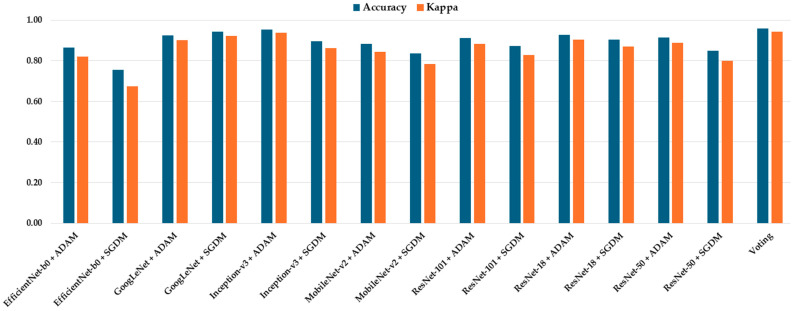
Performance comparison of trained models evaluated on an external independent dataset (rm1000) using accuracy and Kappa metrics.

To evaluate the generalizability and potential clinical applicability of the proposed models, all trained CNN models and the ensemble voting model were tested on an independent external dataset (rm1000) in Table 6. Notably, the ensemble voting model outperformed all individual CNN models across most evaluation metrics, achieving the highest accuracy (0.958) and Kappa score (0.944), thereby demonstrating superior robustness and cross-dataset generalizability (Figure 7). Among the individual models, GoogLeNet + SGDM, Inception-v3 + ADAM, and ResNet-18 + SGDM consistently exhibited strong performance across all tumor categories.

These findings underscore the effectiveness of the proposed optimizer-diverse ensemble strategy in enhancing predictive stability and diagnostic accuracy. Furthermore, the results of this cross-dataset evaluation affirm the reproducibility and clinical relevance of the developed framework, supporting its potential utility in real-world diagnostic settings.

### 5.3. Evaluating the Proposed Method Against Related Works

Table 7 presents a comparative analysis of recent CNN-based studies on brain tumor classification using MRI imaging. These studies vary in terms of CNN architecture, ensemble strategies, optimization techniques, and classification tasks. Most works targeted classification across three or four tumor types, including glioblastoma, meningioma, pituitary adenoma, and “no tumor” categories, with sample sizes ranging from two to four classes and a variety of MRI datasets.

Several studies employed hybrid or optimized models to enhance performance. Notably, Albalawi et al. [2] and Hassan and Ghadiri [4] reported accuracies of 99.0% and 99.16%, respectively, using custom CNNs and EfficientNetV2 combined with statistical techniques. Ait Amou et al. [21] introduced Bayesian optimization for CNNs and achieved a high accuracy of 98.7%, while El Amoury et al. [23] employed PSO-optimized CNNs to reach 99.2%. Among ensemble approaches, Aurna et al. [10] applied a two-stage ensemble achieving 99.13%, and Tandel et al. [20] employed majority voting with explainable AI (XAI), yielding 98.47% accuracy. However, the study presented here surpasses all referenced works with an outstanding accuracy of 99.80%, leveraging a majority voting ensemble across 14 CNN variants. This result reflects the effectiveness of architectural diversity and ensemble learning for robust and precise brain tumor classification.

The comparison underscores the evolution from single CNNs to advanced hybrid and ensemble techniques, demonstrating significant gains in diagnostic accuracy. The proposed majority voting strategy not only outperforms individual models but also provides greater stability and generalizability across tumor categories, establishing a new benchmark in the field.

## 6. Conclusions

### 6.1. Summary of Findings

This study conducted a comprehensive evaluation of seven convolutional neural network (CNN) architectures for multiclass brain tumor classification using T1-weighted contrast-enhanced MRI images. Each model was trained with two different optimization algorithms—SGDM and ADAM—resulting in 14 distinct classifiers. Performance metrics included overall accuracy, Kappa coefficient, class-wise true positive and precision rates, confusion matrices, and ROC curves.

Individually, GoogLeNet and Inception-v3 trained with the ADAM optimizer achieved the highest internal test accuracies (0.987) and Kappa scores (0.983 and 0.980, respectively). However, the proposed majority voting ensemble, which aggregated predictions across all 14 trained models—demonstrated superior performance, achieving a near-perfect accuracy of 0.998 and a Kappa coefficient of 0.997. Class-wise AUC values exceeded 0.997, underscoring the ensemble’s balanced and robust predictive capabilities.

To further assess generalizability, all models were evaluated on an independent external dataset (rm1000), where the ensemble continued to outperform individual CNNs, achieving an accuracy of 0.958 and Kappa score of 0.944. These results affirm the ensemble method’s robustness across datasets and its potential for real-world clinical application. Overall, the integration of architectural and optimizer diversity through majority voting substantially improved classification stability and diagnostic precision in complex neuroimaging tasks.

### 6.2. Limitations and Future Work

Despite the strong classification performance achieved by the proposed majority voting ensemble, several limitations should be acknowledged, which also outline promising directions for future research.

First, although the primary training dataset was well-curated and publicly available, it may not fully capture the heterogeneity found in real-world clinical scenarios. To partially address this, we validated our models on an independent external dataset (rm1000) that differs in image distribution and acquisition conditions. The ensemble model maintained high performance, thereby demonstrating strong generalizability. Nevertheless, further evaluation using multi-center, multi-vendor, and multi-protocol MRI data remains essential to confirm robustness across diverse clinical environments.

Second, the current framework is limited to T1-weighted contrast-enhanced MRI sequences. Incorporating multimodal MRI inputs such as T2-weighted, FLAIR, or diffusion-weighted imaging (DWI) could enhance diagnostic accuracy, especially in cases with ambiguous or atypical presentations.

Third, the model functions as a “black box,” which may hinder clinical trust and interpretability. To address this concern, we have begun integrating Grad-CAM visualizations to highlight discriminative regions utilized by CNNs during classification. Future work will focus on embedding more comprehensive explainable AI (XAI) methods to provide greater transparency and clinician confidence in model predictions.

In addition, extending the classification task beyond tumor types such as incorporating tumor grading, predicting therapeutic response, or estimating patient prognosis—would significantly broaden the model’s clinical utility. Integrating lesion segmentation as a preprocessing step could also refine tumor localization and improve classification accuracy.

Finally, practical deployment in clinical settings would require integration into hospital Picture Archiving and Communication Systems (PACS) or Radiology Information Systems (RIS). This should be coupled with human-in-the-loop verification and prospective clinical trials to validate real-world performance and usability. Addressing these directions will help transition the proposed framework from research innovation to clinically impactful diagnostic support tools.

## Figures and Tables

**Table 1:** Overview of selected CNN architectures for MRI brain tumor classification.

CNN	No. of Layers	Parameters (MB)	Default Input Size	Key Advantages
ResNet-18 [11]	18	11.7	224 × 224	Lightweight, fast training, good for small datasets
ResNet-50 [11]	177	25.6	224 × 224	Deeper network with residual connections for feature reuse
ResNet-101 [11]	347	44.6	224 × 224	Strong performance on complex tasks due to depth
GoogLeNet [12]	22	7.0	224 × 224	Inception modules for multi-scale feature extraction
EfficientNet-b0 [13]	290	5.3	224 × 224	Parameter-efficient, high accuracy with fewer resources
MobileNet-v2 [14]	154	3.5	224 × 224	Optimized for speed and mobile deployment
Inception-v3 [15]	315	23.9	299 × 299	High accuracy with reduced computation

**Table 2:** Classification performance of CNN models using SGDM and ADAM optimizers on brain tumor testing dataset.

CNN	Optimizer	TP1	TP2	TP3	TP4	Pre1	Pre2	Pre3	Pre4	Accuracy	Kappa
ResNet-18	SGDM	0.986	0.964	0.970	0.976	0.960	0.966	0.980	0.995	0.974	0.965
ResNet-18	ADAM	0.983	0.983	0.982	0.988	0.980	0.981	0.980	0.995	0.984	0.978
GoogLeNet	SGDM	0.997	0.974	0.992	0.990	0.976	0.993	0.984	0.996	0.987	0.983
GoogLeNet	ADAM	0.953	0.974	0.972	0.986	0.980	0.971	0.912	0.995	0.971	0.960
EfficientNet-b0	SGDM	0.900	0.898	0.933	0.917	0.897	0.868	0.918	0.960	0.910	0.877
EfficientNet-b0	ADAM	0.959	0.951	0.984	0.967	0.949	0.940	0.970	0.996	0.963	0.949
MobileNet-v2	SGDM	0.910	0.905	0.924	0.925	0.905	0.859	0.920	0.981	0.915	0.885
MobileNet-v2	ADAM	0.965	0.967	0.963	0.954	0.950	0.935	0.978	0.993	0.962	0.949
Inception-v3	SGDM	0.967	0.931	0.926	0.962	0.934	0.923	0.950	0.990	0.949	0.931
Inception-v3	ADAM	0.991	0.975	0.994	0.993	0.978	0.986	0.990	0.997	0.987	0.983
ResNet-50	SGDM	0.939	0.893	0.929	0.888	0.887	0.851	0.912	0.991	0.909	0.877
ResNet-50	ADAM	0.987	0.969	0.974	0.983	0.977	0.968	0.986	0.988	0.979	0.971
ResNet-101	SGDM	0.932	0.897	0.877	0.897	0.879	0.818	0.954	0.988	0.903	0.869
ResNet-101	ADAM	0.983	0.966	0.974	0.985	0.959	0.969	0.990	0.998	0.977	0.969
Voting		0.996	0.997	0.998	1.000	0.999	0.996	1.000	0.997	0.998	0.997

**Table 3:** Confusion matrix, class-wise true positive rate (TP), false positive rate (FP), and precision for optimal CNN model (GoogLeNet with SGDM optimizer) on entire dataset.

Class	Glioblastoma	Meningioma	No Tumor	Pituitary	TP	FP
Glioblastoma	904	18	3	1	0.976	0.024
Meningioma	2	927	0	5	0.993	0.007
No Tumor	1	4	492	3	0.984	0.016
Pituitary	0	3	1	897	0.996	0.004
Precision	0.953	0.974	0.972	0.986	Accuracy	0.987
FP	0.047	0.026	0.028	0.014	Kappa	0.983

**Table 4:** Confusion matrix, class-wise true positive rate (TP), false positive rate (FP), and precision for optimal CNN model (Inception-v3 with ADAM optimizer) on entire dataset.

CNN	Glioblastoma	Meningioma	No Tumor	Pituitary	TP	FP
Glioblastoma	906	19	1	0	0.978	0.022
Meningioma	6	921	2	5	0.986	0.014
No Tumor	1	3	495	1	0.990	0.010
Pituitary	1	2	0	898	0.997	0.003
Precision	0.953	0.974	0.0972	0.986	Accuracy	0.987
FP	0.047	0.026	0.028	0.014	Kappa	0.983

**Table 5:** Confusion matrix, class-wise true positive rate (TP), false positive rate (FP), and precision for Voting Schema on entire dataset.

CNN	Glioblastoma	Meningioma	No Tumor	Pituitary	TP	FP
Glioblastoma	922	4	0	0	0.996	0.004
Meningioma	0	931	0	3	0.997	0.003
No Tumor	1	0	499	0	0.998	0.002
Pituitary	0	0	0	901	1.000	0.000
Precision	0.953	0.974	0.0972	0.986	Accuracy	0.998
FP	0.047	0.026	0.028	0.014	Kappa	0.997

**Table 6:** Comparative performance of CNN models tested on an independent external dataset (rm1000).

Model	TP1	TP2	TP3	TP4	Pre1	Pre2	Pre3	Pre4	Accuracy	Kappa
EfficientNet-b0 + ADAM	0.791	0.829	0.985	0.845	0.840	0.765	0.909	0.933	0.865	0.820
EfficientNet-b0 + SGDM	0.632	0.651	0.970	0.803	0.714	0.708	0.703	0.893	0.754	0.673
GoogLeNet + ADAM	0.841	0.945	0.992	0.930	0.970	0.899	0.908	0.931	0.926	0.901
GoogLeNet + SGDM	0.933	0.868	0.996	0.968	0.888	0.951	0.956	0.969	0.942	0.923
Inception-v3 + ADAM	0.925	0.916	0.988	0.977	0.955	0.946	0.980	0.927	0.953	0.937
Inception-v3 + SGDM	0.880	0.818	0.986	0.896	0.889	0.830	0.886	0.980	0.897	0.862
MobileNet-v2 + ADAM	0.847	0.824	0.983	0.871	0.853	0.822	0.895	0.956	0.883	0.844
MobileNet-v2 + SGDM	0.787	0.756	0.984	0.827	0.850	0.760	0.804	0.935	0.837	0.783
ResNet-101 + ADAM	0.902	0.827	0.974	0.938	0.853	0.902	0.957	0.925	0.912	0.882
ResNet-101 + SGDM	0.822	0.811	0.974	0.860	0.838	0.742	0.910	0.978	0.871	0.828
ResNet-18 + ADAM	0.899	0.875	0.991	0.939	0.912	0.913	0.935	0.948	0.928	0.903
ResNet-18 + SGDM	0.888	0.837	0.995	0.891	0.875	0.885	0.878	0.975	0.903	0.871
ResNet-50 + ADAM	0.902	0.848	0.987	0.915	0.891	0.879	0.941	0.941	0.915	0.887
ResNet-50 + SGDM	0.866	0.749	0.980	0.815	0.833	0.759	0.821	0.984	0.850	0.800
Voting	0.943	0.934	1.000	0.948	0.938	0.921	0.980	0.986	0.958	0.944

**Table 7:** Comparison of CNN-Based brain tumor classification studies in MRI imaging.

Authors	Year	Method	Task	Classes	Accuracy
AlTahhan et al. [1]	2023	Hybrid AlexNet-KNN	Classification	4	98.60%
Albalawi et al. [2]	2024	Multi-layer CNN	Classification	4	99.00%
Nahiduzzaman et al. [3]	2025	PDSCNN + RRELM	Classification	4	99.20%
Hassan & Ghadiri [4]	2025	EfficientNetV2	Classification	3	99.16%
Gupta et al. [5]	2024	Custom CNN	Classification	2	94.00%
Kusuma & Reddy [6]	2025	SegNet + Bi-LSTM	Segmentation & Classification	4	98.00%
Disci et al. [7]	2025	Transfer Learning (Xception, etc.)	Classification	4	98.73%
Ali et al. [8]	2025	Classic CNN + ResNet50	Classification	3	99.88%
Al-Azzwi & Nazarov [9]	2023	Stacked Ensemble (VGG19, etc.)	Classification	2	96.60%
Aurna et al. [10]	2022	Two-Stage Ensemble CNN	Classification	4	99.13%
Iqbal et al. [16]	2024	FusionNet (Statistical + CNN)	Classification	2	97.53%
Elhadidy et al. [17]	2025	Swin Transformer + EfficientNet	Classification	4	98.72%
Abirami et al. [18]	2025	AKO-Shepard CNN	Classification & Detection	3	93.60%
Alsubai et al. [19]	2022	CNN-LSTM	Classification	3	99.10%
Tandel et al. [20]	2025	Majority Voting + XAI	Classification	4	98.47%
Ait Amou et al. [21]	2022	CNN + Bayesian Optimization	Classification	3	98.70%
Deepa et al. [22]	2023	Hybrid Optimization + DRN	Segmentation & Classification	3	92.10%
El Amoury et al. [23]	2025	PSO-Optimized CNN	Classification	4	99.20%
Huang et al. [25]	2025	ResNet50V2 + SE Blocks	Classification	4	98.40%
Jarria & Wesley [26]	2025	Fruit Bee Optimized CNN	Classification	3	92.60%
da Costa Nascimento et al. [27]	2025	YOLO + LLM	Detection & Classification	2	98.00%
Chandraprabha et al. [28]	2025	ViT + CNN	Classification	4	99.64%
Afzal et al. [29]	2025	ResNet18 + CART-ANOVA	Classification	4	98.05%
This Study	2025	Majority Voting Ensemble	Classification	4	99.80%

## Data Availability

The MRI brain tumor datasets used in this study are publicly available on Kaggle. The training data were obtained from https://www.kaggle.com/datasets/prathamgrover/brain-tumor-classification accessed on 5 November 2024, and the independent testing data were sourced from https://www.kaggle.com/datasets/rm1000/brain-tumor-mri-scans accessed on 4 July 2025.
